# Why Amphibians Are More Sensitive than Mammals to Xenobiotics

**DOI:** 10.1371/journal.pone.0007699

**Published:** 2009-11-04

**Authors:** Angelo Quaranta, Vito Bellantuono, Giuseppe Cassano, Claudio Lippe

**Affiliations:** 1 Department of Animal Production, University of Bari, Italy; 2 Department of General and Environmental Physiology, University of Bari, Italy; Universidad Europea de Madrid, Spain

## Abstract

Dramatic declines in amphibian populations have been described all over the world since the 1980s. The evidence that the sensitivity to environmental threats is greater in amphibians than in mammals has been generally linked to the observation that amphibians are characterized by a rather permeable skin. Nevertheless, a numerical comparison of data of percutaneous (through the skin) passage between amphibians and mammals is lacking. Therefore, in this investigation we have measured the percutaneous passage of two test molecules (mannitol and antipyrine) and three heavily used herbicides (atrazine, paraquat and glyphosate) in the skin of the frog *Rana esculenta* (amphibians) and of the pig ear (mammals), by using the same experimental protocol and a simple apparatus which minimizes the edge effect, occurring when the tissue is clamped in the usually used experimental device.

The percutaneous passage (P) of each substance is much greater in frog than in pig. LogP is linearly related to logK_ow_ (logarithm of the octanol-water partition coefficient). The measured P value of atrazine was about 134 times larger than that of glyphosate in frog skin, but only 12 times in pig ear skin. The FoD value (P_frog_/P_pig_) was 302 for atrazine, 120 for antipyrine, 66 for mannitol, 29 for paraquat, and 26 for glyphosate.

The differences in structure and composition of the skin between amphibians and mammals are discussed.

## Introduction

The evidence that the sensitivity to environmental threats is higher in amphibians than in mammals has been generally linked to the observation that amphibians are characterized by a rather permeable skin [1], [2]. Previous studies have investigated the absorption of xenobiotics in amphibians [3]–[6] and mammals [7]–[9], but a numerical comparison of the results so far reported is lacking. In order to obtain quantitative information supporting the general belief that amphibians are more sensitive than mammals to contaminants, in this investigation we have measured the percutaneous (through the skin) passage of two test molecules (mannitol and antipyrine) and three heavily used herbicides (atrazine, paraquat and glyphosate) in frog (amphibians) and pig ear skin (the most appropriate model of human skin) [10], by using the same experimental protocol.

Dramatic declines in amphibian populations have been described all over the world since the 1980s [11]. The IUCN Red List Categories of Vulnerable, Endangered, or Critically Endangered species [12] includes 32.5% of the total number of amphibian species but only 12% and 23% of birds and mammals, respectively [13], [14]. Furthermore, the absolute number of individuals is also decreasing in 2468 amphibian species (43.2%), while the population is stable for 1552 species (27.2%) and it is increasing in only 28 cases (0.5%) [15].

Causes of biodiversity loss include overexploitation and habitat loss, caused either by climate change [16] or by direct human activity [17]. However, extinctions have also been reported in pristine habitats where the above effects should not occur [18]. In particular, high threat risk has been correlated with large amphibian species living in small geographic areas with pronounced seasonality in temperature and precipitation [19].

Amphibians are more sensitive than birds and mammals to xenobiotics mainly for two reasons. Since they spend the first and the second part of their life in aquatic and terrestrial environments, respectively, they have to face the threats present in both habitats. Secondly, amphibian skin is highly permeable because it is physiologically involved in gas, water, and electrolyte exchange with the environment.

**Figure 1:**
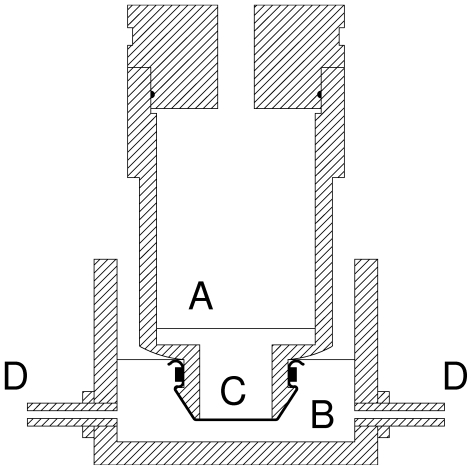
The device used to measure the percutaneous passage of substances in frog and pig ear. (A) Donor chamber. (B) Receptor chamber. (C) Skin. (D) Externally supplied air. The tissue was fixed with a plastic tie.

To obtain information about percutaneous absorption, *in vitro* techniques are used which have been reviewed elsewhere [20], [21]. The experimental device usually utilized is a diffusion cell consisting of a donor chamber and a receptor chamber between which the skin is clamped. However, the permeability of the tissue under investigation is affected by the compression exerted at its edge by clamping and this effect has been referred to as edge damage. For example, in frog skin the edge damage caused the measured values of permeability to urea and sodium to increase by 7- and 20.8 fold above their nonedge-damaged values [22]. For this reason, we have used an alternative simple apparatus, minimizing the edge effect (Figure 1).

## Materials and Methods

^14^C-labelled 6-chloro-4-N-ethyl-2-N-propan-2-yl-1,3,5-triazine-2,4-diamine (atrazine), 2-(phosphonomethylamino)acetic acid (glyphosate), D-mannitol, and 1-methyl-4-(1-methylpyridin-1-ium-4-yl)pyridin-1-ium dichloride (paraquat) were obtained from Sigma-Aldrich (Milano, Italy) and 4-iodo-1,5-dimethyl-2-phenylpyrazol-3-one (antipyrine) from Perkin Elmer NEN (Monza (Mi), Italy).

Excised ears of one year old pigs, obtained from a local abattoir, were immediately transported to our laboratory and after removing any extraneous subcutaneous tissue, were immediately used.

Adult *Rana esculenta*, grown in the Naples region (Italy) and kept in the animal care facility of our Department according to the protocols approved by the Italian Ministry for Scientific Research, were sacrificed and their ventral skin was removed. A pig or frog skin was mounted on the specially constructed static diffusion cell, shown in Figure 1, consisting of a donor and an aerated receptor chamber, containing respectively 7 and 20 ml of a solution with the following composition (in mM): 120 NaCl, 5 KCl, 2.5 CaCl_2_, 1 MgSO_4_, 0.001 NaN_3_, 15.6 Na_2_HPO_4_, pH 7.4 (with HCl) for pig skin experiments; in the case of frog skin the composition of the solution was (in mM): 112 NaCl, 5 KCl, 1 CaCl_2_, 2.5 NaHCO_3_, pH 8.1. The ^14^C-labelled tested substance (0.1 µCi/ml) was only present in the donor compartment (“infinite dose assay” [23]), facing the internal surface of the skin; so the flux from body side to external medium was measured. After a 24 h (pig ear) or 6 h (frog) period, ad room temperature (20–22°C), samples were collected from the receptor chamber and analyzed for radioactivity.The permeability coefficient P (cm/h) was calculated from P = J/(A C) where J (cpm/h) is the total flux, A is the diffusion area (2.1 cm^2^ in our case), and C (cpm/cm^3^) is the concentration of the test substance in the donor compartment.

In this paper we used the logK_ow_ (logarithm of octanol-water partition coefficient) values measured by other authors in the cases of antipyrine, atrazine, mannitol [24], and glyphosate [25] or calculated by us with the Estimation Program Interface Suite™ software (http://www.epa.gov/oppt/exposure/pubs/episuitedl.htm) in the case of paraquat. Data are shown as mean ± standard error values from 14 and 7 experiments with atrazine, 9 and 12 with antipyrine, 11 and 15 with mannitol, 10 and 10 with paraquat, 17 and 8 with glyphosate in frog and pig, respectively.

## Results

**Figure 2:**
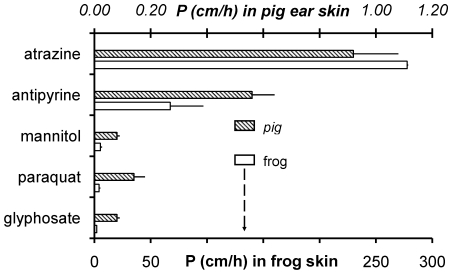
The percutaneous passage (unidirectional flux through the skin) of atrazine, antipyrine, mannitol, paraquat, and glyphosate in pig ear (dashed symbols, upper scale) and frog (open symbols, lower scale). Data are shown as mean ± standard error values from 7, 12, 15, 10, 8 and 14, 9, 11, 10, 17 and experiments in pig and frog, respectively, following 24 h (pig) or 6 h (frog) incubation at room temperature (20–22°C).

*In vitro* methods make it possible to measure the unidirectional flux (passage) of a molecule through a biological tissue and provide a convenient way of investigating the basic principles underlying percutaneous absorption. In this study, starting from the flux measurements through the skin of frog and pig ear, we have calculated the permeability coefficient (P) for a panel of substances (Figure 2). Data showed that the P values of two test molecules (mannitol and antipyrine) and three heavily used herbicides (atrazine, paraquat and glyphosate) were much higher in frog skin (open bars and lower scale) than in pig skin (upper scale).

**Figure 3:**
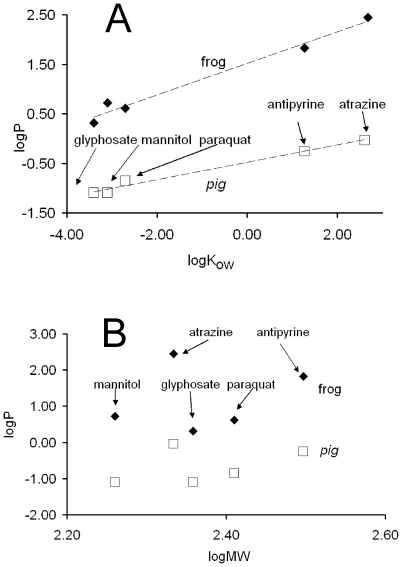
(A) Dependence of the measured logP on logK_ow_ (hydrophobicity) of the substance or (B) on logMW (molecular weight). Data from Figure 2 were used.

We then verified whether the set of measured P values depends on the hydrophobicity of the investigated molecules and/or their molecular weight. In Figure 3A logP of atrazine, antipyrine, mannitol, paraquat, and glyphosate are presented as a function of logK_ow_ (where K_ow_ is the octanol-water partition coefficient). The K_ow_ of a chemical substance is the ratio at equilibrium of its concentrations in the two phases of a mixture of octanol and water and indicates the degree of the hydrophobicity of that substance. When two substances are compared, the drug with a higher K_ow_ value is more hydrophobic and more permeable through hydrophobic compartments such as the lipid component of the cell membrane.

In Figure 3A, in the cases of frog and pig ear skin, the measured logP values linearly depend on logK_ow_ (hydrophobicity) of the substances, as expected. The coefficient of determination (R^2^) values, obtained by using a linear regression procedure, were 0.9797 and 0.9833 for frog and pig, respectively. In Figure 3B the logP values have also been plotted versus logMW (molecular weight); in this case the R^2^ values (0.0534 and 0.183 for frog and pig, respectively) indicate that no correlation exists between the two variables.

**Figure 4:**
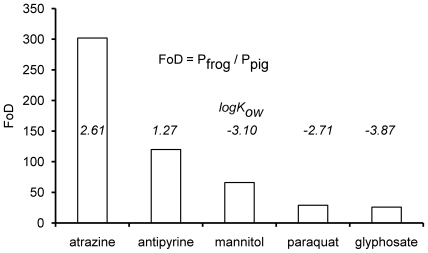
The Factors of Difference (FoD = P_frog_/P_pig_) and log K_ow_ values of atrazine, antipyrine, mannitol, paraquat, glyphosate. Data from Figure 2 were used.

We have so far shown that: a) the flux of each substance was always much higher in frog than in pig ear skin (Figure 2); b) in particular the ratio P_atrazine_/P_glyphosate_ was 134 in frog and 12 in pig; c) the line interpolating the frog data is steeper than the other (Figure 3A). These observations suggest that the structure of the skin is different in frog and pig. In order to find further support to our suggestion, we then calculated the factors of difference (FoD values) using the following expression: FoD value  = P_frog_/P_pig_ [7]. In Figure 4, these FoD values are reported and range from 302 (atrazine) to 26 (glyphosate); over each bar the logK_ow_ of the substance is also reported; evidently, the more hydrophobic the substance, the higher its FoD.

## Discussion

The skin in aquatic and semi-aquatic amphibians is generally characterized by a permeability higher than in any other order of vertebrates because it is physiologically engaged in respiration and in the regulation of internal concentration of water and ions [26]. From an evolutionary point of view, the development of an integument limiting the cutaneous water loss has been an important step in the radiation of vertebrates in terrestrial environments [27].

For this paper we measured the flux of five substances through the skin of frog and pig ear. The following order of permeability values was found in frog: atrazine > antipyrine > mannitol > paraquat > glyphosate, while in pig it was: atrazine > antipyrine > paraquat > glyphosate and mannitol. Moreover, the logP values were dependent on logK_ow_ (the octanol-water partition coefficient) but independent of logMW (molecular weight). Starting from the measured flux values, we also calculated the factors of difference (FoD), using the expression: FoD = P_frog_/P_pig_, [7]. FoD values are 302, 120, 66, 29, and 26 for atrazine, antipyrine, mannitol, paraquat, and glyphosate, respectively. These values indicate that the measured fluxes were higher in frog than in pig by at least one order of magnitude, and that the more hydrophobic the substance, the higher the FoD value. The transepithelial transfer of molecules occurs either through the space between adjacent cells or through cells. These two pathways are referred to as paracellular and transcellular, respectively. The selectivity of the paracellular barrier is controlled by the specificity of the tight junctions (closely associated areas of two adjacent cells [28]) and mainly affects the diffusion of hydrophilic molecules. On the other hand, the selectivity of the transcellular transfer is shaped by the apical and basolateral plasma membrane; such a process occurs in the cases of hydrophobic molecules or when a specific transporter is present (for example aquaporines for water).

We measured higher permeability values in frog than in pig ear; this fact is obviously caused by the differences between the skin of the two animals. The skin is a specialized epithelium that isolates the organism from the environment and prevents the loss of endogenous material. It consists of four layers: the hypodermis, dermis, viable epidermis and stratum corneum. The latter is the closest to the surface and the thinnest region, and represents the barrier to percutaneous absorption [27], [29]. Generally, the permeability of an epithelium is proportional to its thickness and the stratum corneum is roughly 10 times thicker in pig than in frog being 20 µm and ≈2 µm (*Rana pipiens*), respectively [30], [31]. Nevertheless, the fact that in our case the FoD values for atrazine and antipyrine were 302 and 120, while the stratum corneum thickness accounts for only one order of magnitude, leads us to suggest that other explanations must exist, possibly the structure of the stratum corneum as well as the composition and geometry of barrier lipids.

While in dehydration-sensitive amphibians the stratum corneum consists of one or two cell layers, in mammals it is multilayered and provides a structural template for sealing lipids that perform a water barrier function.

Concerning the lipid composition of the skin, in amphibians a strict correlation between the properties of extracted lipids and rates of evaporative water loss has not been reported [32]. As opposed to amphibians, in mammals the stratum corneum can be described as a brick wall, where the bricks are the corneocytes (cell remnants of the terminally differentiated keratinocytes found in viable epidermis) and the mortar is the abundant intercellular lipid [33] organized in sheets, termed multiple stacked lipidic lamellae, the composition of which is quite different from that of cell membranes [34]. In man and pig, intercellular lipid has a unique composition, consisting of roughly equimolar concentrations of free fatty acids, cholesterol and ceramides [34]. Keratinocytes secrete the content of lamellar bodies, which are Golgi derived organelles, in the extracellular domain where lipids are enzymatically converted into multilayered structures that occlude the extracellular spaces among corneocytes. In amphibians, intracellular organelles similar to mammalian lamellar bodies have not been described. Moreover, the layered complexes of lipid and keratin, which are present in reptiles, birds and mammals, are absent in amphibians, with the interesting exception of the “cocoons” occurring in some species during estivation [28].

Finally, another parameter strongly influencing the body concentration of environmental xenobiotics is the skin/body ratio which, for evolutionary reasons, is maximized in amphibians (exchanging gases, water and ions with the environment through the skin) and minimized in mammals (in order to lose less heat). In this paper we have shown that a xenobiotic can diffuse into a frog (amphibian) one or two orders of magnitude faster (depending on its hydrophobicity) than into a pig. We have discussed the structural differences between the integument of the two animals under investigation. In this paper we also show the simplicity of the measurement and prediction of the rate by which a xenobiotic diffuses through the skin. We artlessly hope that in future these studies will precede investigations about the negative effect of substances once they are already present in the environment [35].
